# Decidualized human decidual stromal cells inhibit chemotaxis of activated T cells: a potential mechanism of maternal-fetal immune tolerance

**DOI:** 10.3389/fimmu.2023.1223539

**Published:** 2023-08-23

**Authors:** Tatiana Llorca, Maria Jose Ruiz-Magaña, Rocio Martinez-Aguilar, Olga María García-Valdeavero, Lucia Rodríguez-Doña, Ana Clara Abadia-Molina, Carmen Ruiz-Ruiz, Enrique G. Olivares

**Affiliations:** ^1^ Instituto de Biopatología y Medicina Regenerativa, Centro de Investigación Biomédica, Universidad de Granada, Granada, Spain; ^2^ Departamento de Bioquímica y Biología Molecular III e Inmunología, Universidad de Granada, Granada, Spain; ^3^ Unidad de Gestión Clínica Laboratorios, Hospital Universitario Clínico San Cecilio, Granada, Spain

**Keywords:** chemotaxis, decidual stromal cells, decidualization, Th1 cells, Tc cells

## Abstract

**Background:**

Numerous lines of evidence confirm that decidual stromal cells (DSCs) play a key role in maternal–fetal immune tolerance. Under the influence of progesterone and other hormones, the DSCs go through a process of differentiation (decidualization) during normal pregnancy. In mice, DSCs inhibit the expression of chemokines that attract abortigenic Th1 and Tc cells to the decidua. We have studied this phenomenon in humans.

**Methods:**

We established human DSC lines and decidualized these cells *in vitro* with progesterone and cAMP. We determined the expression of the chemokines *CXCL9*, *CXCL10* and *CXCL11*, whose receptor CXCR3 is expressed by Th1 and Tc cells, in undifferentiated DSCs and decidualized DSCs by qRT-PCR. Activated CD3+CXCR3+ cells, including CD4+ Th1 cells and CD8+ Tc cells, were induced *in vitro*. The migration capacity of these activated lymphocytes was investigated in Transwell chambers with conditioned media from undifferentiated and decidualized DSCs.

**Results:**

We demonstrated that *CXCL9* was not expressed by DSCs, whereas the expression of *CXCL10* and *CXCL11* was inhibited in decidualized cells. Conditioned media from decidualized cells significantly inhibited the migration of Th1 and Tc cells. We found that decidualized cells secrete factors of MW less than 6000–8000 Da, which actively inhibit the chemotaxis of these lymphocytes.

**Discussion:**

These results confirm in humans that decidualization of DSCs inhibits the expression by these cells of chemokines that attract Th1 and Tc cells and induces the secretion by DSCs of factors that inhibit the chemotaxis of these lymphocytes, thus preventing the arrival of abortigenic T cells in the decidua.

## 1 Introduction

Pregnancy can be regarded as a semi-allogeneic transplant in the mother that is not rejected under normal conditions because of the many mechanisms that support the mother’s immunological tolerance to the fetus (1). In early publications on this topic, one of these proposed mechanisms was the inhibition of Th1 cell activity and Tc cell cytotoxicity against the fetus because of the predominance of Th2 cell responses in normal pregnancy (2), whereas abortion was associated with Th1 cell responses (3, 4). The fact that Th1 cells play a physiological role in certain stages of pregnancy such as implantation and delivery (5, 6), together with the discovery of new Th cell types, challenged the Th1-Th2 paradigm, and some authors considered it too simplistic (5) or controversial (1); thus, new models of Th lymphocyte-dependent immune response in pregnancy have been proposed (7). However, the fact that pregnant women are more susceptible to viral infections that rely on a Th1 cell-associated immune response, together with the amelioration of Th1 cell-associated autoimmune diseases, supports the notion that except for the beginning and end of pregnancy, when inflammation and Th1 cells play a physiological role, the Th1 response is inhibited during normal pregnancy (8–12).

The decidua (pregnant endometrium), as the maternal part of the placenta in direct contact with the fetal trophoblast, controls maternal–fetal immune interactions. Decidual stromal cells (DSCs), the main cellular component of human decidua, play a key role in the normal development of pregnancy through their immunoregulatory activities (13). DSCs originate from fibroblastic perivascular precursors (preDSCs) that differentiate into decidualized DSCs (dDSCs) under the influence of progesterone (P4) and other pregnancy hormones. In the process of decidualization, dDSCs leave the vessels toward the extravascular space, become more rounded, and secrete prolactin (PRL) and other proteins such as IL-15 (13). Some authors consider that DSCs are the result of differentiation of endometrial stromal cells (EnSCs) (14). However, we have shown that precurssor and decidualized cells exist both in the decidua and in the endometrium (preEnSCs and dEnSCs) (13). Although they are the same cells in different environments (non-pregnancy and pregnancy), we have observed that DSCs have a greater capacity for decidualization than EnSCs (15), so it is important to distinguish one type of uterine stromal cell from the other. During the first trimester, the decidua faces the immunological challenge posed by fetal tissues, making the decidua an appropriate setting to study the mechanisms of maternal–fetal immunological tolerance.

Perivascular preDSCs are in a privileged position to control leukocyte chemotaxis from the peripheral blood into the decidua. In fact, human preDSCs have a chemotactic effect on peripheral blood NK cells and monocytes (16, 17). Nancy et al., working with pregnant mice, demonstrated epigenetic silencing of T cell-attracting inflammatory chemokine genes (CXCR3 ligands CXCL9, CXCL10 and CXCL11, and CCR5 ligand CCL5) in DSCs. Because CXCR3 and CCR5 are expressed by Th1 and Tc cells, this would imply that these abortigenic effector T cells do not accumulate within the decidua during pregnancy, thus providing a mechanism of maternal–fetal immune tolerance. These authors also indicated that decidualization of DSCs may favor this regulatory function (18). This mechanism of immune tolerance, however, has not yet been studied in humans. In the present study we investigated the expression by human DSCs of chemokines that attract activated T cells, as well as the chemotactic capacity of DSCs in these lymphocytes.

## 2 Materials and methods

### 2.1 Samples

Samples were obtained by suction curettage from elective vaginal terminations of first trimester pregnancies (6–11 weeks) in women 20–30 years old at the Clinica Ginegranada in Granada. Use of any medication or any infectious, autoimmune, or other systemic or local disease were considered exclusion criteria. None of the abortions were pharmacologically induced. Blood samples for peripheral blood lymphocyte (PBL) isolation were collected from healthy volunteers 20–35 years old at Hospital Universitario Clínico San Cecilio in Granada.

### 2.2 Monoclonal antibodies

The mAbs used are listed in **Supplementary Table S1**.

### 2.3 Tumor cell lines

The human tumor cell lines used were THP-1 monocytic leukemia and Jurkat human leukemia T cells. Both lines were cultured in RPMI 1640 medium (Gibco, Carlsbad, CA) supplemented with 100 IU/mL penicillin, 100 μg/mL streptomycin (Sigma-Aldrich, St. Louis, MO), and 10% fetal calf serum (FCS) (Invitrogen, Grand Island, NY).

### 2.4 Isolation and culture of preDSC lines

For this study, we established 12 preDSC lines from decidua (each from a different sample) according to the method described by Kimatrai and colleagues (19). Briefly, decidua samples were minced between two scalpels in a small volume of PBS, and the suspension was mixed with a 5 mg/mL solution of collagenase V (Sigma-Aldrich) for 30 min at 37°C before centrifugation at 425 g for 10 min. The pellet was suspended in PBS and centrifuged on Ficoll-Paque (Sigma-Aldrich) at 600 g for 20 min. Decidual cells were collected from the interface, suspended in PBS and washed. Cells were incubated overnight in culture flasks at 37°C with 5% CO_2_ in Opti-MEM (Invitrogen) supplemented with 3% FCS (Invitrogen), 100 IU/mL penicillin, 100 μg/mL streptomycin and 0.25 μg/mL amphotericin (Sigma-Aldrich) to allow adherent cells to attach to the flask. The medium was then replaced to discard nonadherent cells in the supernatant, and then changed twice a week thereafter. Adherent cells exhibited a uniform fibroblastic morphology and covered the entire surface of the 25-cm^2^ culture flask after 1–3 weeks. Cells were split with 0.25% trypsin-EDTA solution (Sigma-Aldrich) when they were 90–100% confluent. After 3–8 weeks, preDSCs proliferate and outgrow macrophages, resulting in pure lines of preDSCs. Established cell lines were used between 3 and 8 weeks after collection (up to 5 passages). The maternal origin of each preDSC line was confirmed with aneuploid analysis by comparison with the corresponding trophoblasts obtained from the same sample, using short tandem repeat markers of chromosomes 13, 18, 21, X and Y, and quantitative-fluorescent PCR (Devyser AB, Hägersten, Sweden). Aneuploid samples were discarded. The preDSC lines consisted of a uniform, adherent, highly purified cell population of fibroblastic morphology with a characteristic antigenic profile (16, 19), in which the cells were CD45-negative and almost all of them expressed CD29 and the endometrial stromal cell marker CD10 (20).

### 2.5 Decidualization

To induce decidualization, preDSC lines were treated with 300 nM P4 and 500 μM dibutyryl cAMP (Sigma-Aldrich) for 8 days. Decidualization was confirmed by observing changes in cell morphology from a fibroblastic shape in preDSCs to a round shape in dDSCs, by light microscopy, and by determining PRL production with an electrochemiluminescence immunoassay (Roche, Indianapolis, IN) according to the manufacturer’s instructions (17).

### 2.6 Conditioned media

Conditioned media (CM) from undifferentiated pre-DSCs (preDSC-CM) and dDSCs (dDSC-CM) were collected from cultures after 8 days, centrifuged at 1000 g, sterile filtered, and stored frozen until use. As a control, we used complete culture medium. To determine the characteristics of active factors of the CM, they were heated to 60°C for 45 min. These CM were also dialyzed (Membrane Tubing 6000–8000 Da molecular weight cut-off [MWCO], Fisher Scientific, Madrid, Spain) against Opti-MEM for 24 h.

### 2.7 Activation of peripheral blood lymphocytes

PBLs were separated by Ficoll-Paque density centrifugation (Sigma-Aldrich) and incubation with RPMI 1640 medium supplemented with 10% FCS, 100 IU/mL penicillin and 100 μg/mL streptomycin for 1 h at 37°C to eliminate adherent cells. For activation, PBLs were then cultured at 2 × 10^6^ cells/ml with 5 μg/mL phytohemagglutinin (Sigma-Aldrich) and 1 μg/mL anti-CD28 (eBioscience, San Diego, CA) for 20 h. After washing, the cells were incubated for an additional 6 days in complete RPMI medium supplemented with 25 U/mL rIL-2 (National Institute of Health AIDS Reagent Program, Rockville, MD). Activated lymphocytes expressed CXCR3 (>95%), including CD4+ Th1 cells (20–41%), CD8+ Tc (46–65%), and CD56+ NK cells (6–14%).

### 2.8 Migration assay

Migration assays were performed in 24-Transwell chemotaxis chambers with a pore size of 3 μM (Corning Incorporated, New York, NY). The bottom of each well was covered with 600 μL of control fresh complete Opti-MEM, or preDSC-CM, or dDSC-CM. Then 2.5 × 10^5^ cells in 100 μL complete Opti-MEM were added to each insert. After 24 h at 37°C, the inserts were removed, and the bottom cells were counted. The number of migrated cells was determined with a hemocytometer by light microscopy, and cell viability was determined by trypan blue exclusion. The results were expressed as the number of migrated cells or percentage cell migration, calculated with the formula: (number of migrated cells/total number of cells) × 100. Before and after migration, cells were subjected to flow cytometry to determine subpopulations. The percentage results obtained from the subpopulations were applied to the total number of cells previously quantified by light microscopy to obtain the absolute number of cells in each subpopulation.

### 2.9 Chemotaxis blockade

Chemotactic activity was blocked with anti-CXCR3 mAb (anti-human CD183, Biolegend), using mouse IgG1 (Biolegend) as a blocking control. Antibodies at different concentrations were incubated with 1.25 × 10^6^/mL activated PBL at room temperature for 45 min and washed in PBS before the migration assay.

### 2.10 Flow cytometric analysis

PBL were washed with PBS supplemented with 0.5% BSA and 2 mM EDTA. One hundred microliters of a suspension of PBL at 10^6^ cells/mL was incubated with 5 μL of the appropriate mAb or the isotype control for 30 min at 4°C in the dark. The cells were then washed, suspended in 0.5 mL PBS, and analyzed in a fluorescence-activated cell sorter (FACSCalibur Cytometer, BD Biosciences, San Jose, CA). PBL were gated in an SSC-H and FSC-H dot plot to select lymphocytes (**Supplementary Figure S1A**). Viability staining was carried out with propidium iodide (PI) (Sigma). The percentage of antibody-positive cells was calculated in comparison to the appropriate isotype control. For double labeling, the same procedure was followed as for single labeling but a second mAb with a different fluorescent marker was added. For indirect labeling, APC- or FITC-labeled goat anti-mouse Ig was added after the first mAb.

### 2.11 Reverse transcription polymerase chain reaction

Total RNA was extracted from DSCs or PBL with the TRIzol reagent (Thermo Fisher Scientific, Waltham, MA). cDNA was synthesized at an RNA starting concentration of 0.5 μg with the Access RT-PCR System kit (Promega, Madison, WI) and oligo-dT primers according to the manufacturer’s protocol. Conventional RT-PCR was carried out in a 2720 Thermal Cycler (Applied Biosystems, Foster City, CA) by mixing cDNA samples with the PCR Mastermix 2x kit (Promega) and the appropriate primers. PCR products were separated by size on 1.5% agarose gels stained with Gel Red (Biotium, Fremont, CA), including a 100-bp DNA ladder (Promega) in each run. The bands were then quantified with Adobe Photoshop CS3 software (Adobe, San Jose, CA). Quantitative PCR (RT-qPCR) was done in a Quantstudio 3 Real-time PCR system (Applied Biosystems), using the appropriate primers and FastStart Universal SYBR Green Master (Merck Spain, Madrid) according to the manufacturer’s instructions. The cut-off was set at 35. Primer sequences are listed in **Supplementary Table S2**.

### 2.12 Statistical analysis

The results are expressed as the mean ± SD of at least three separate assays with different DSC lines. All experiments were performed in triplicate. The unpaired two-tailed t-test with Welch’s correction was used. P *<* 0.05 values were considered significant.

## 3 Results

### 3.1 Effects of DSC decidualization on the expression of chemokines that attract activated T cells

**Figure 1:**
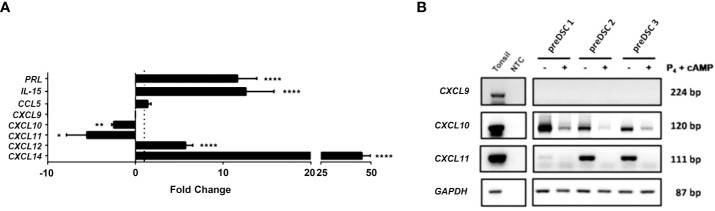
Expression of different genes in DSCs before and after decidualization. **(A)** RT-qPCR data were normalized to the expression of the housekeeping gene glyceraldehyde-3-phosphate dehydrogenase (*GAPDH*) and calculated with the 2^− ΔCT^ formula. Fold change (FC) represents dDSC values relativized to preDSC values. To preserve graph symmetry, FC values less than 1 (negative fold change, NFC) were represented with the formula NFC= -1/FC. **(B)** RT-PCR analysis. Human tonsil was used as a positive control. NTC: Negative control (nuclease-free water). *P < 0.05, **P < 0.01, ****P < 0.0001. Number of DSC lines tested: n = 3.

To determine the effects of decidualization of DSCs on the expression of chemokines CXCL9, CXCL10, CXCL11 and CCL5, which are involved in the chemotaxis of activated T lymphocytes, the expression of these genes and those associated with decidualization was studied by RT-qPCR and confirmed by RT-PCR in preDSCs and dDSCs. Decidualization increased the expression of genes associated with this differentiation process, such as *PRL, IL15, CXCL12* and *CXCL14* as assessed by RT-qPCR (**Figure 1A**) (16, 17, 21). Regarding the expression of chemokines that attract activated T cells, it was observed with RT-qPCR that DSCs did not express *CXCL9* or change their expression of *CCL5* upon decidualization; however, dDSCs significantly downmodulated the expression of *CXCL10* and *CXCL11* (**Figure 1A**). These latter results were also confirmed by RT-PCR (**Figure 1B**).

### 3.2 Chemotactic activity of DSCs on activated PBLs

**Figure 2:**
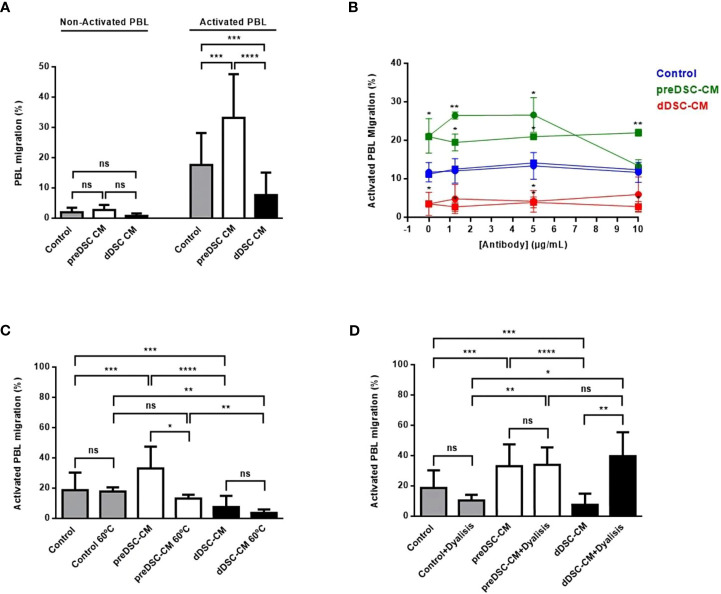
Chemotaxis of activated lymphocytes under the effects of DSC-CM. **(A)** Effects of preDSC-CM, dDSC-CM and Control medium (Opti-MEM with 3% FCS) on migration in Transwell plates of nonactivated and activated PBL. Migration was calculated as (number of migrated cells/total number of cells) × 100. **(B)** Blocking of the chemotactic activity of DSC-CM in activated PBL with an anti-CXCR3 mAb (•-•) or control mouse IgG1 (◼-◼). **(C)** Effect of heating pre-DSC-CM and dDSC-CM on activated PBL chemotaxis. **(D)** Effect of dialysis of pre-DSC-CM and dDSC-CM on activated PBL chemotaxis. ns, not significant, *P < 0.05, **P < 0.01, ***P < 0.001, ****P < 0.0001. In **(B)** the values were compared with those of their respective controls (blue). Number of DSC lines tested: **(A)** (n = 6), **(B–D)** (n = 3).

To determine the effects of factors secreted by preDSCs and dDSC on the chemotaxis of activated lymphocytes, migration assays were performed with preDSC-CM and dDSC-CM. Decidual stromal cell-CM showed no activity in nonactivated PBL. PreDSC-CM significantly increased the migration of activated lymphocytes, but dDSC-CM significantly inhibited this migration (**Figure 2A**). We used complete culture medium as a control; however, complete culture medium containing P4 and cAMP for dDSC-CM was not used, because considering that both factors enter the cells, it is likely that dDSC-CM no longer contain them. Furthermore, we tested controls with P4 and cAMP and found no significant differences compared to controls without P4 and cAMP (**Supplementary Figure S2**). The fact that blocking CXCR3 (the CXCL10 and CXCL11 receptor) with a mAb abrogated the migration induced by preDSC-CM demonstrated that CXCL10 and CXCL11 were responsible for this activity. However, this mAb showed no effect on the inhibitory activity of dDSC-CM. Blocking control mouse IgG1 had no effect on either type of chemotactic activity of CM. The high expression of CXCR3 by activated T lymphocytes (**Supplementary Figure S1A**) probably explains why blockade of this receptor by anti-CXCR3 was achieved only at the highest concentration of this antibody (**Figure 2B**). Heating dDSC-CM did not affect its ability to inhibit migratory activity, whereas heating reduced the chemotactic effect of preDSC-CM, probably due to chemokine denaturation (**Figure 2C**). However, dialysis had the opposite effect: the migratory activity induced by dialyzed preDSC-CM was maintained (MW of CXCL10 and CXCL11 were higher than 8000 Da), whereas the inhibitory activity of dialyzed dDSC-CM disappeared, showing that the MW of the inhibitory factors was lower than that of the MWCO (**Figure 2D**). Taken together, these results demonstrate that CXCL10 and CXCL11 are the chemokines secreted by preDSCs that are responsible for the increased chemotaxis of activated lymphocytes. On the other hand, dDSCs secrete thermostable factors with MW lower than 6000–8000 Da, which inhibit this chemotaxis.

### 3.3 Characteristics of migrated activated PBL

**Figure 3:**
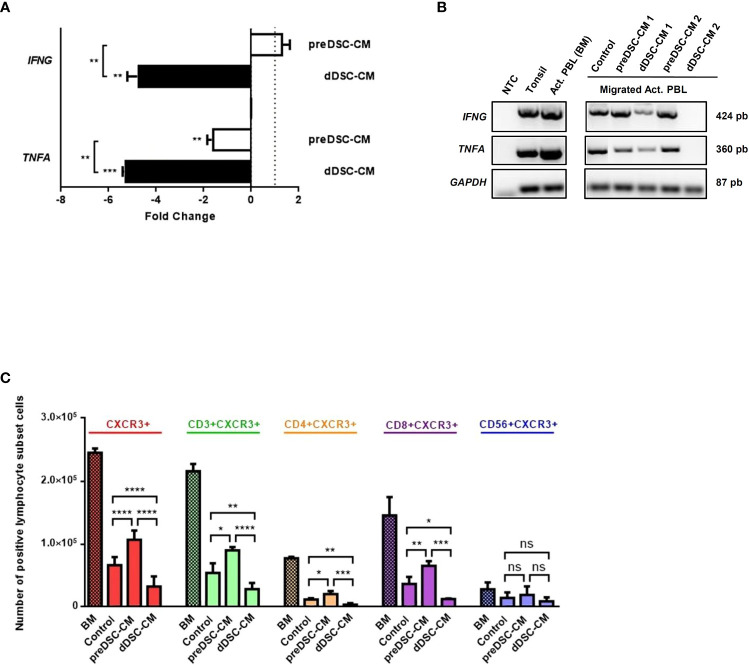
Effects of DSC-CM on migrated activated lymphocytes. **(A)** RT-qPCR analysis of the expression of *IFNG* and *TNFA* by migrated lymphocytes. Fold change represents the normalized RT-qPCR values from migrated activated lymphocytes exposed to CM, relativized against values of migrated activated lymphocytes exposed to Control medium. **(B)** RT-PCR analysis. **(C)** Chemotactic effects of DSC-CM on different activated lymphocyte subpopulations. Crossed bars represent the numbers of activated lymphocytes before migration (BM). Non-crossed bars are the numbers of migrated lymphocytes. Human tonsil was used as a positive control. NTC, Negative control (nuclease-free water). ns, not significant, *P < 0.05, **P < 0.01, ***P < 0.001, ****P < 0.0001. Number of DSC lines tested: **(A, B)** (n = 3), **(C)** (n = 6).

To study the characteristics of migrated lymphocytes, we determined the expression of Th1 chemokines *IFNG* and *TNFA* by RT-PCR and RT-qPCR, and analyzed the different activated lymphocyte subpopulations by flow cytometry. dDSC-CM downmodulated the expression of Th1 cytokines *IFNG* and *TNFA* in migrated activated lymphocytes. PreDSC-CM also inhibited *TNFA* expression, although they had no effect on *IFNG* expression (**Figures 3A, B**). Flow cytometry analysis of the migrated activated lymphocyte subpopulations after exposure to CM showed that the effects of preDSC-CM (activation) or dDSC-CM (inhibition) on migration affected activated T cells (CD3+CXCR3+), including Th1 (CD4+CXCR3+) and Tc cells (CD8+CXCR3+), but had no significant effect on activated NK cells (CD56+CXCR3+) (**Figure 3C**; **Supplementary Figure S3**). The effect of DSC-CM on the migration of Jurkat cells (a tumor Th cell line) was also examined. In contrast to activated T cells, preDSC-CM showed no significant effect on Jurkat cells, probably because of the much lower expression of CXCR3 by these tumor cells than by activated T cells (**Supplementary Figure S1A**). However, like activated T cells, dDSC-CM inhibited the migration of Jurkat cells while enhancing the migration of the monocyte line THP-1 (**Supplementary Figure S1B**), as previously reported (17). Taken together, these results show that preDSCs increase the chemotaxis of Th1 and Tc cells, whereas dDSCs inhibit the chemotaxis of these lymphocytes, but have no effect on activated NK cells. dDSCs also inhibit the expression of Th1 chemokines on the migrated activated lymphocytes.

## 4 Discussion

Recent lines of experimental evidence confirm the involvement of Th1 cells in abortion. Thus, in normal pregnancy, different mechanisms of maternal–fetal immune tolerance inhibit the differentiation to Th1 cells or the arrival of these cells to the decidua (8–10, 12, 18). Nancy et. al., in their work with a mouse model, demonstrated the reduced capacity of DSCs to produce Th1 and Tc cell chemoattractants (CXCL9, CXC10, CXCL11 and CCL5), and these authors suggested that decidualization was the cause of the inability to produce these chemokines (18). Similarly, the present study with human cells shows that CXCL9 was not detected in either preDSCs or dDSCs, and the expression of CXCL10 and CXCL11 was inhibited after decidualization. However, our experimental approach differs from that of Nancy et. al: they studied the expression of chemokines in cytokine-activated mouse DSCs, while we analyzed the direct effect of decidualization on this expression in human DSCs. Because CXCL9, CXCL10 and CXCL11 are ligands of CXCR3 (22) – the receptor expressed by Th1, Tc, and NK cells (23) – our findings are consistent with those of Nancy et al., and also show that decidualization of DSCs inhibits the expression of chemokines that attract these activated lymphocytes. However, we did not find a significant change in the expression of CCL5 in dDSCs. In this context, it should be noted that CCL5 is involved in relevant functions during normal human pregnancy: it promotes the physiological invasion of trophoblasts into the decidua (24), participates in the recruitment of Treg cells, and favors decidualization (25). Furthermore, whereas CXCL9, CXCL10 and CXCL11 attract activated lymphocytes with abortogenic effects, CCL5 receptors are expressed on a wide variety of immune cells (26), some of which (e.g. monocytes) reach the decidua in normal pregnancy (27).

Because preDSCs produced CXCL10 and CXCL11, which attract activated T lymphocytes, decidualization of DSCs, which inhibits the expression of these chemokines, would appear to control this chemotactic activity. Nevertheless, the absence of CXCL9 together with the downmodulation of CXCL10 and CXCL11 expression in dDSCs do not explain the significant inhibition of activated lymphocyte migration induced by dDSC-CM, since the effect of this downmodulation alone on migration would be similar to that induced by the control with culture medium and not significantly lower as seen in the present results. This significant decrease, i.e. less migration than under control conditions, therefore suggests that in addition to inhibiting CXCL10 and CXCL11 expression, dDSCs actively inhibit chemotaxis. One possible explanation for this active inhibition is the production by dDSCs of factors that induce lymphocyte apoptosis, as observed in some tumor cell lines (17); however, DSCs were shown to have no effect, or even to protect lymphocytes from apoptosis (17, 28). Another possibility is that dDSCs secrete one or more factors that actively inhibit the migration of activated lymphocytes, i.e. T cell chemotaxis-inhibiting factors (TCIFs). Although we do not yet know the nature of these factors, we have shown that they have a MW below 6000–8000 Da and that they are thermostable. Furthermore, we were able to verify that TCIFs are not associated with exosomes, since the extraction of exosomes from dDSC-CM did not alter the effects of the medium on the migration of activated lymphocytes (**Supplementary Figure S4**). Factors detected in the maternal–fetal interphase, some of which are associated with decidualization, may be related to TCIFs (29–32). Further research is underway to identify the biochemical characteristics of TCIFs.

**Figure 4:**
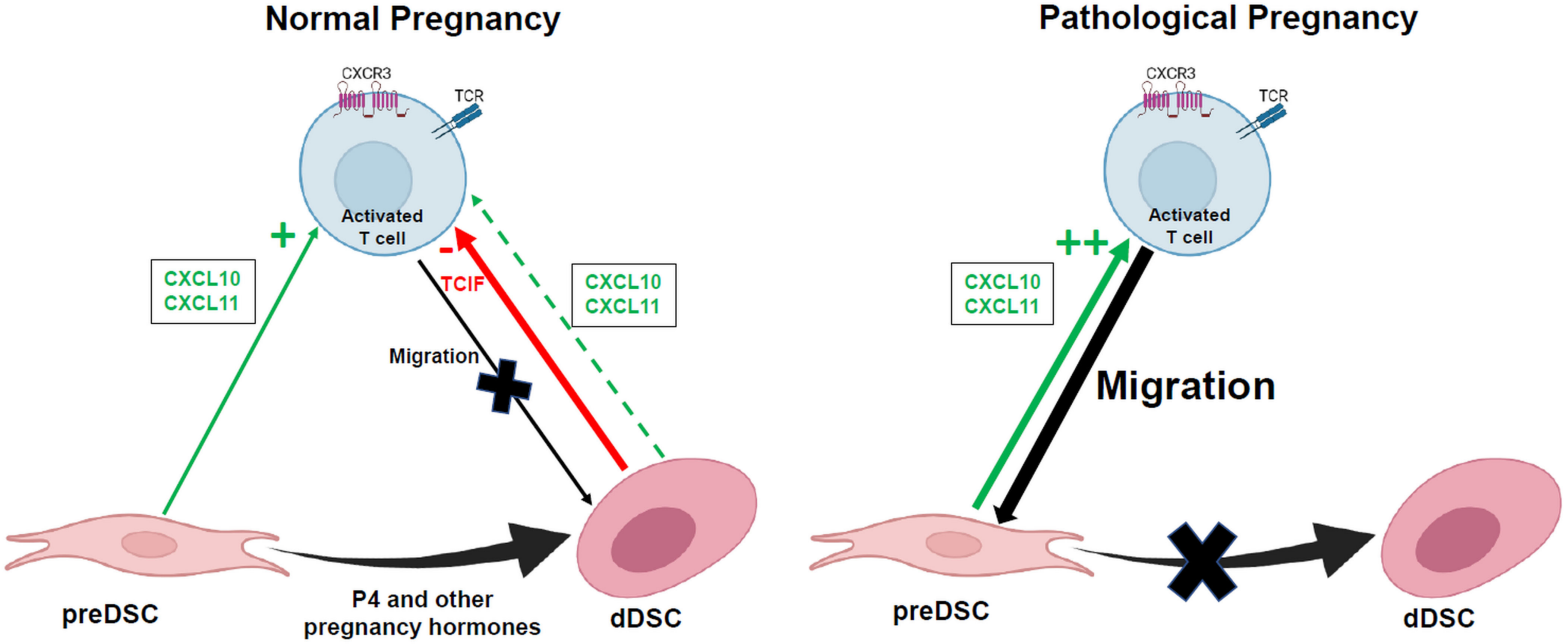
Hypothetical action of factors secreted by DSCs on activated T cell migration to the decidua. TCIF, T cell chemotaxis-inhibiting factor. Figure created in BioRender.com.

Both the activating (pre-DSCs) and inhibitory (dDSCs) effect on chemotaxis was exerted on Th1 and Tc. However, activated NK cell chemotaxis was not significantly affected by preDSCs or dDSCs. The inhibitory effect of dDSC on the chemotaxis of the THP-1 monocytic line was also not observed, suggesting that TCIFs act primarily on activated T cells. Furthermore, TNF and IFNγ – Th1 cytokines produced by these T lymphocytes – were downmodulated by the effect of dDSCs, showing that these stromal cells not only inhibited Th1 and Tc cell chemotaxis, but also inhibited the expression of Th1 cytokines in these lymphocytes. Taken together, our results confirm the existence of a mechanism of immune tolerance associated with decidualization of DSCs that appears to be involved in controlling the arrival of activated T lymphocytes in the decidua. Evidence that supports this mechanism is based on 1) the lack of expression of CXCL9 and inhibition of the expression by dDSCs of CXCL10 and CXCL11, chemokines that attract Th1 and Tc cells, 2) the secretion of as yet uncharacterized low-molecular-weight thermostable factors that actively inhibit the chemotaxis of these lymphocytes, and 3) inhibition of the expression of *TNFA* and *IFNG* by migrated lymphocytes. Although this mechanism would operate in normal pregnancy, in other situations such as (for example) infection, inflammatory molecules inhibit DSC decidualization (33), so that the chemotactic effect of preDSCs on Th1 and Tc cells becomes predominant. These T cells are likely to play a role in inducing abortion, a process that is often associated with infection (34) (**Figure 4**). Other pathological processes associated with defective decidualization may also favor the arrival of activated T cells to the decidua. Conversely, the tolerogenic activities of DSCs may explain the therapeutic effects of DSCs on murine immune-mediated abortion (35), and on inflammatory diseases in humans (36). The identification and isolation of TCIFs may be a promising avenue of research in the treatment of autoimmune and Th1-mediated inflammatory diseases.

## Data availability statement

The original contributions presented in the study are included in the article/**Supplementary Material**. Further inquiries can be directed to the corresponding authors.

## Ethics statement

The studies involving humans were approved by The Research and Ethics Committee of University of Granada. The studies were conducted in accordance with the local legislation and institutional requirements. The participants provided their written informed consent to participate in this study.

## Author contributions

TL and MR-M contributed to the study design, execution, data analysis and critical discussion. RM-A, OG-V and LR-D collected samples, and participated in the execution of the study and data analysis. AA-M contributed to the study design, data interpretation and critical discussion. EO and CR-R were responsible for the conception and study design, financial support, data analysis and interpretation, and manuscript writing. All authors contributed to the article and approved the submitted version.

## Supplementary material

Supplementary Figure 1
**(A)** Flow cytometry analysis of the expression of CXCR3 by activated PBL and Jurkat T cells. **(B)** Chemotactic effects of DSC-CM on Jurkat T cells and THP-1 monocytes. **P < 0.01, ***P < 0.001, ****P < 0.0001. Number of DSC lines tested: n = 3.

Supplementary Figure 2 Chemotaxis of activated lymphocytes under the effects of DSC-CM. Complete culture medium and complete culture medium plus P4 and cAMP were used as controls. Number of DSC lines tested: n = 3.

Supplementary Figure 3 Flow cytometry analysis of activated T cells migrated under the effect of DSC-CM. The number of cells in each quadrant is displayed. Number of DSC lines tested: n = 6.

Supplementary Figure 4 Effect of the extraction of exosomes from dDSC-CM on chemotactic activity of activated lymphocytes. Exosomes were extracted by ultracentrifugation. Number of DSC lines tested: n = 3.
