# Supplementary material for: Decidualized human decidual stromal cells inhibit chemotaxis of activated T cells: a potential mechanism of maternal-fetal immune tolerance
Source: Front Immunol. 2023 Aug 23;14:1223539. doi: 10.3389/fimmu.2023.1223539 (PMC10481401; doi:10.3389/fimmu.2023.1223539)
Supplement: Supplementary file 1 [file Table_1.docx]

**Supplementary Table S1**. List of monoclonal antibodies**^1^**

| **Antibody** | **Type** | **Conjugated fluorophore** | **Clone** |
| --- | --- | --- | --- |
| Anti-human CD3 | IgG2a | FITC | HIT3a |
| Anti-human CD4 | IgG1 | FITC | SK3 |
| Anti-human CD8 | IgG1 | PE | SK1 |
| Anti-human CD56 | IgG1 | APC | 5.1H11 |
| Anti-human CD183 (CXCR3) | IgG1 | Purified | G025H7 |
| Mouse IgG2a, κ Isotype Control | IgG2a | FITC | MOPC-173 |
| Mouse IgG1, κ Isotype Control | IgG1 | FITC | MOPC-21 |
| Mouse IgG1, κ Isotype Control | IgG1 | PE | MOPC-21 |
| Mouse IgG1, κ Isotype Control | IgG1 | APC | MOPC-21 |
| Goat anti-mouse IgG | IgG | FITC | - |
| Goat anti-mouse IgG | IgG | APC | - |

**^1^**All monoclonal antibodies were purchased from Biolegend, San Diego, CA

**Supplementary Table S2**. List of primers**^1^**

| **Gene^2^** | **Primer sequence** | **Product size (bp)** | **Tm (°C)** |
| --- | --- | --- | --- |
| Prolactin (PRL) | F: 5’-AAT CTG TTC CGC TGG TGA CT-3’  R: 5’-GAA GTG GGG CAG TCA TTG AT-3’ | 297 | 58,5 |
| IL-15 | F: 5’-CAA ACA ACA GTT TGT CTT CTA ATG G-3’  R: 5’-GGA CAA TAT GTA CAA AAC TCT GCA A-3’ | 114 | 53,5 |
| CCL5 | F: 5’-AAG GAG TAT TTC TAC ACC-3’  R: 5’-CCA AAG AGT TGA TGT ACT-3’ | 124 | 45 |
| CXCL9 | F: 5’-CCT TGA AAG ACC TTA AAC-3’  R: 5’-AGA ACG TTG AGA TTT TC-3’ | 224 | 46 |
| CXCL10 | F: 5’-GAA AGC AGT TAG CAA GGA AAG GTC-3’  R: 5’-ATG TAG GGA AGT GAT GGG AGA GG-3’ | 120 | 60 |
| CXCL11 | F: 5’-TAC AGT TGT TCA AGG CTT CCC-3’  R: 5’-GGG TAC ATT ATG GAG GCT TTC TC-3’ | 111 | 59 |
| CXCL12 | F: 5’-CAA CAC TCC AAA CTG TGC CC-3’  R: 5’-TAG CTT CGG GTC AAT GCA CA-3’ | 79 | 59 |
| CXCL14 | F: 5’-ACG GGT CCA AAT GCA AGT-3’  R: 5’-CGC TCT TGG TGG TGA TGA TAA-3’ | 126 | 55 |
| IFN-γ (RT-PCR) | F: 5’-AGC TCT GCA TCG TTT TGG GT-3’  R: 5’-CGC TTC CCT GTT TTA GCT GC-3’ | 424 | 58 |
| IFN-γ (RT-qPCR) | F: 5’-GAG TGT GGA GAC CAT CAA GGA AG-3’  R: 5’-TAT GCT TTG CGT TGG ACA TTC AAG TC-3’ | 126 | 65 |
| TNF-α (RT-qPCR) | F: 5’-GTC AGA TCA TCT TCT CGA ACC-3’  R: 5’-CAG ATA GAT GGG CTC ATA CC-3’ | 360 | 54 |
| TNF-α (RT-qPCR) | F: 5’-CCC AGG GAC CTC TCT CTA ATC A-3’  R: 5’-GCT ACA GGC TTG TCA CTC GG-3’ | 80 | 60 |
| GAPDH | F: 5’-GCA CCA CCA ACT GCT TAG C-3’  R: 5’-GGC ATG GAC TGT GGT CAT GAG-3’ | 87 | 59 |

**^1^**All primers were purchased from Integrated DNA Technologies (Coralville, IA) except PRL and TNF-α, which were purchased from Instituto de Parasitología y Biomedicina, Granada, Spain.

**^2^**Except as indicated in parentheses, all primers were used for both RT-qPCR and RT-PCR.
