# Supplementary figures and images for: Decidualized human decidual stromal cells inhibit chemotaxis of activated T cells: a potential mechanism of maternal-fetal immune tolerance
Source: Front Immunol. 2023 Aug 23;14:1223539. doi: 10.3389/fimmu.2023.1223539 (PMC10481401; doi:10.3389/fimmu.2023.1223539)

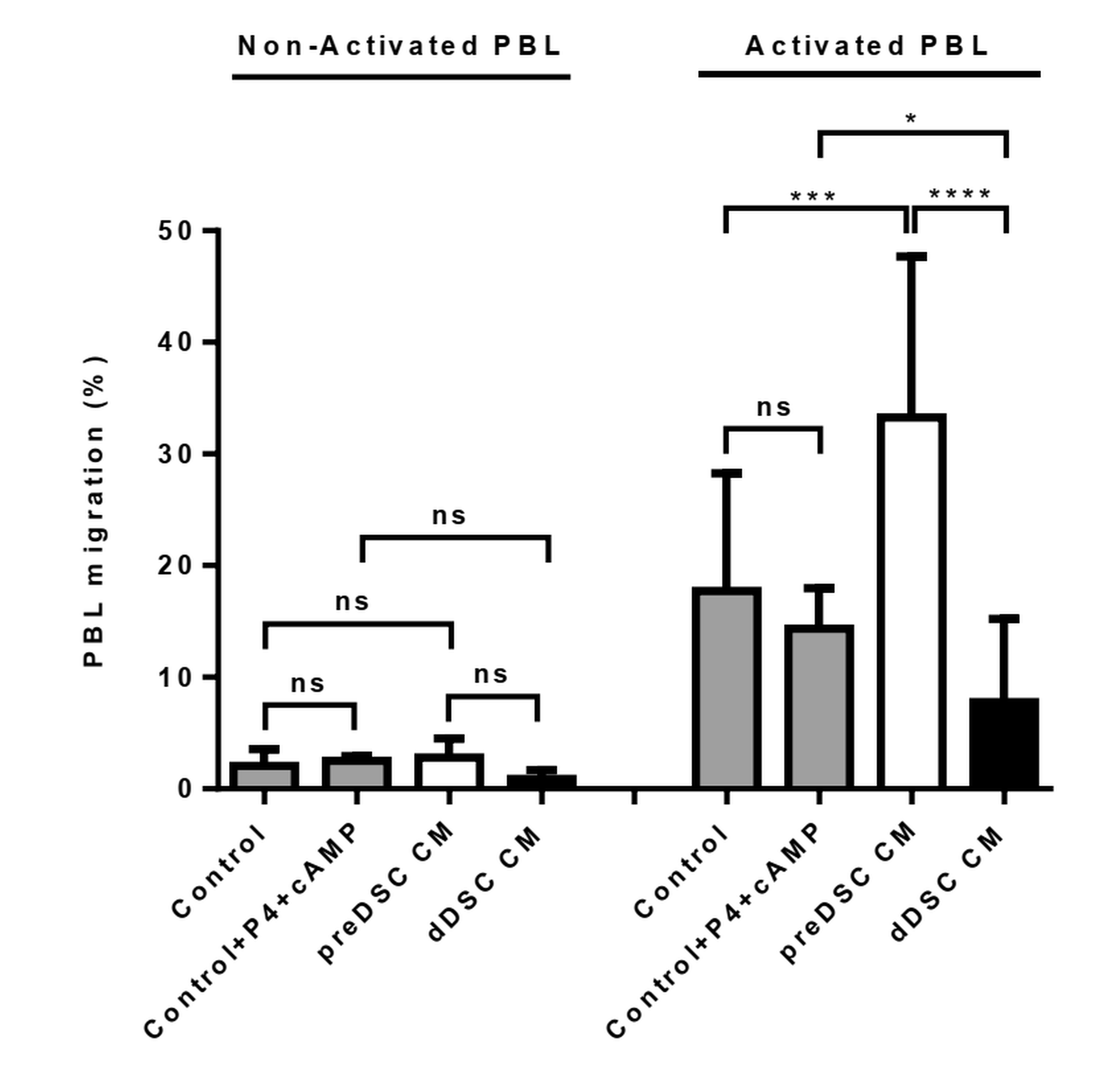

Supplement: Supplementary Figure 2 — Chemotaxis of activated lymphocytes under the effects of DSC-CM. Complete culture medium and complete culture medium plus P4 and cAMP were used as controls. Number of DSC lines tested: n = 3. [file Image_2.jpeg]

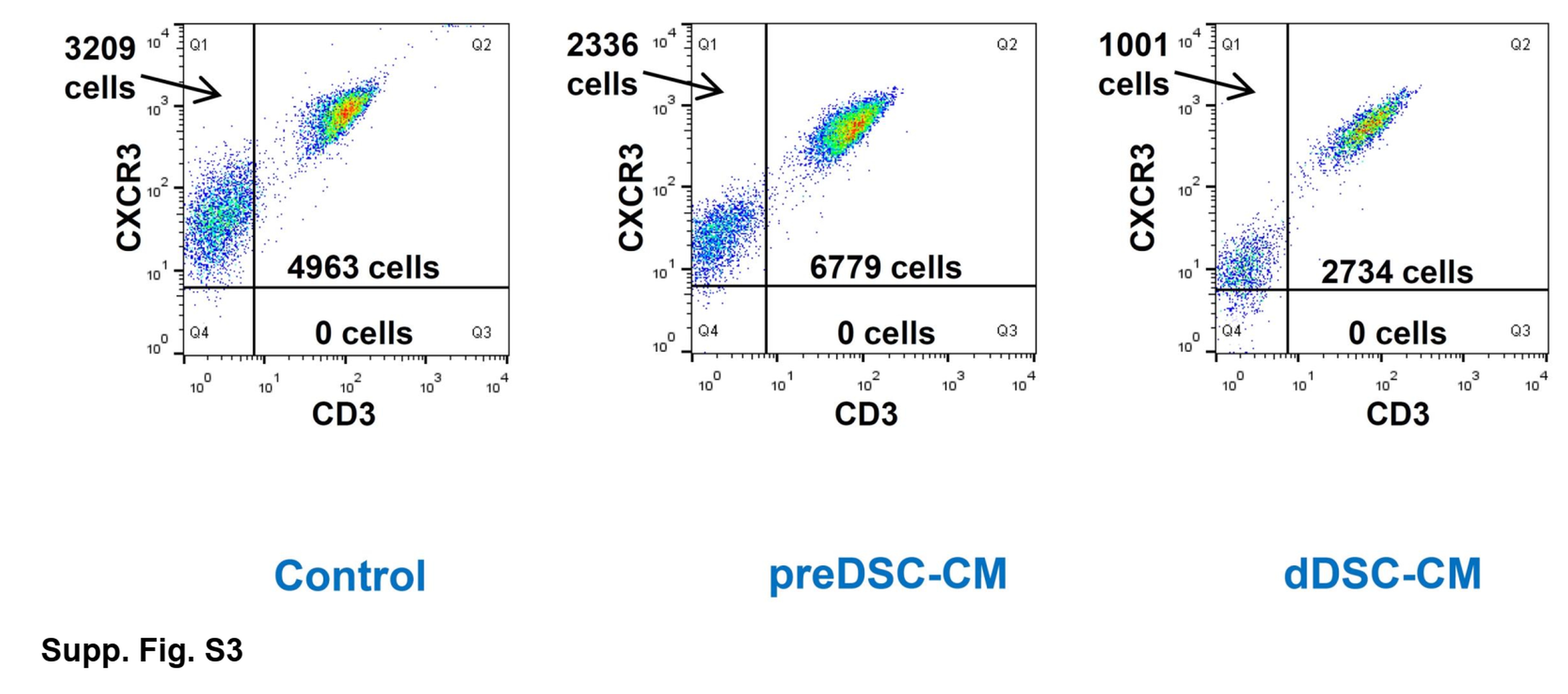

Supplement: Supplementary Figure 3 — Flow cytometry analysis of activated T cells migrated under the effect of DSC-CM. The number of cells in each quadrant is displayed. Number of DSC lines tested: n = 6. [file Image_3.jpeg]

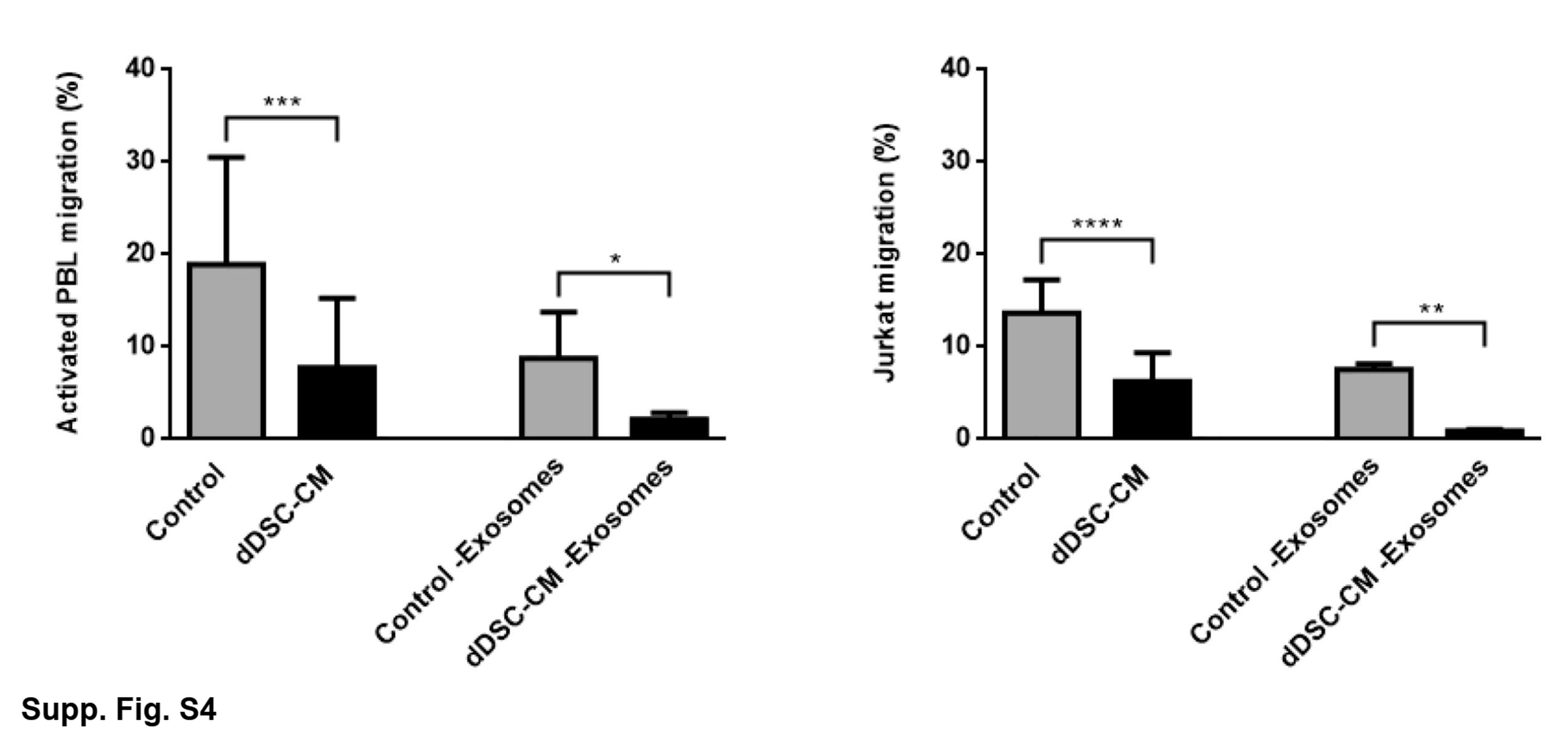

Supplement: Supplementary Figure 4 — Effect of the extraction of exosomes from dDSC-CM on chemotactic activity of activated lymphocytes. Exosomes were extracted by ultracentrifugation. Number of DSC lines tested: n = 3. [file Image_4.jpeg]
